# Melatonin prevents postovulatory oocyte aging and promotes subsequent embryonic development in the pig

**DOI:** 10.18632/aging.101252

**Published:** 2017-06-26

**Authors:** Tao Wang, Ying-Ying Gao, Li Chen, Zheng-Wen Nie, Wei Cheng, Xiaoyan Liu, Heide Schatten, Xia Zhang, Yi-Liang Miao

**Affiliations:** ^1^ Institute of Stem Cell and Regenerative Biology, College of Animal Science and Technology & College of Veterinary Medicine, Huazhong Agricultural University, Wuhan 430070, China; ^2^ Key Laboratory of Agricultural Animal Genetics, Breeding and Reproduction, Huazhong Agricultural University, Ministry of Education, Wuhan 430070, China; ^3^ The Cooperative Innovation Center for Sustainable Pig Production, Wuhan 430070, China; ^4^ Reproductive Medicine Centre, Affiliated Hospital of Qingdao University, Yuhuangding Hospital of Yantai, Yantai, Shandong 264000, China; ^5^ Department of Veterinary Pathobiology, University of Missouri, Columbia, MO 65211, USA

**Keywords:** oocyte aging, melatonin, pig, embryo development

## Abstract

Oxidative stress is known as a major contributing factor involved in oocyte aging, which negatively affects oocyte quality and development after fertilization. Melatonin is an effective free radical scavenger and its metabolites AFMK and AMK are powerful detoxifiers that eliminate free radicals. In this study, we used porcine oocytes to test the hypothesis that melatonin could scavenge free radicals produced during oocyte aging, thereby maintaining oocyte quality. We compared reactive oxygen species levels, apoptosis levels, mitochondrial membrane potential ratios, total glutathione contents and expression levels in fresh, aged and melatonin-treated aged porcine oocytes and observed the percentage of blastocyst formation following parthenogenetic activation. We found that melatonin could effectively maintain the morphology of oocytes observed in control oocytes, alleviate oxidative stress, markedly decrease early apoptosis levels, retard the decline of mitochondrial membrane potential and significantly promote subsequent embryonic development in oocytes aged for 24 hr *in vitro*. These results strongly suggest that melatonin can prevent postovulatory oocyte aging and promote subsequent embryonic development in the pig, which might find practical applications to control oocyte aging in other mammalian species including humans to maintain the quality of human oocytes when performing clinical assisted reproductive technology.

## INTRODUCTION

It is well known that oocyte aging induces several functional changes and these changes affect oocyte quality and subsequent embryo development after fertilization. The changes include cortical granule exocytosis [1], zona pellucida (ZP) hardening [2], chromosome and spindle anomalies [3], decreased fertilization rates [4] and abnormal and/or retarded development of embryos/fetuses [5]. Therefore, it is important to determine the mechanisms that are implicated in oocyte aging, in order to develop strategies to delay oocyte aging and increase the time that may be needed to manipulate oocytes to perform assisted reproductive technologies (ARTs).

Oxidative stress has been shown to induce the above changes and cause a decline in critical cell cycle factors such as maturation-promoting factor (MPF) during oocyte aging [6–8]. Free radicals consisting of reactive oxygen species (ROS) and reactive nitrogen species (RNS) are the main products of oxidative reactions. Post-ovulatory aging proceeds with the concomitant excessive accumulation of oxidative stress, and aged oocytes are prone to undergo apoptosis or retarded embryonic development following fertilization [9, 10]. As the major “energy generators”, mitochondria have an important role in maintaining proper function and survival of oocytes, following ovulation. However, it is widely known that mitochondria are the major ROS generator and mitochondria are particularly susceptible to ROS-induced damage [11], which in turn results in decreased ATP synthesis and mitochondria membrane potential and triggers oxidative stress and early apoptosis [12, 13].

As of now, a number of candidates have been included in a list of components that block oxidative stress. Dithiothreitol (DTT) might protect oocytes by preventing oxidation of free thiol groups and/or altering a redox-independent signaling pathway that mediates cellular fragmentation and death [14]. Further assays demonstrated that caffeine could delay oocyte aging by maintaining normal meiotic spindle morphology [15, 16]. Sugiyama et al reported that resveratrol increased mitochondrial DNA copy numbers and the ATP content in oocytes and improved the developmental ability of oocytes [17]. Melatonin had been shown to be a specific hormone-involved in regulating biological rhythms. However, more studies showed that melatonin was an effective free radical scavenger [9, 18, 19] and promoted embryonic development [20–22]. Furthermore, melatonin, as a lipophilic molecule, is able to exert its direct free radical scavenging action through a membrane receptor-modulated pathway [23]. Moreover, it was revealed that melatonin along with its metabolites, AFMK and AMK, are powerful detoxifiers that are able to eliminate free radicals [24].

Melatonin was shown to prevent oocyte aging and extend the window for optimal fertilization *in vitro* in the mouse [9]. However, how specifically melatonin inhibits oocyte aging has not yet been investigated. We therefore employed the porcine mammalian system that has been shown in many aspects to be very similar to human germ cells and propose the hypothesis that melatonin can scavenge free radicals produced during oocyte aging and maintain the quality of oocytes. In the present study, we determined an optimal supplementation concentration of melatonin for porcine postovulatory aged oocytes, and we determined ROS levels, apoptosis levels, mitochondrial membrane potential ratio, total glutathione (T-GSH) contents and expression levels in fresh, aged and melatonin-treated aged porcine oocytes and the percentage of blastocyst formation following parthenogenetic activation.

## RESULTS

### Effects of different concentrations of melatonin on delaying oocyte aging

**Figure 1:**
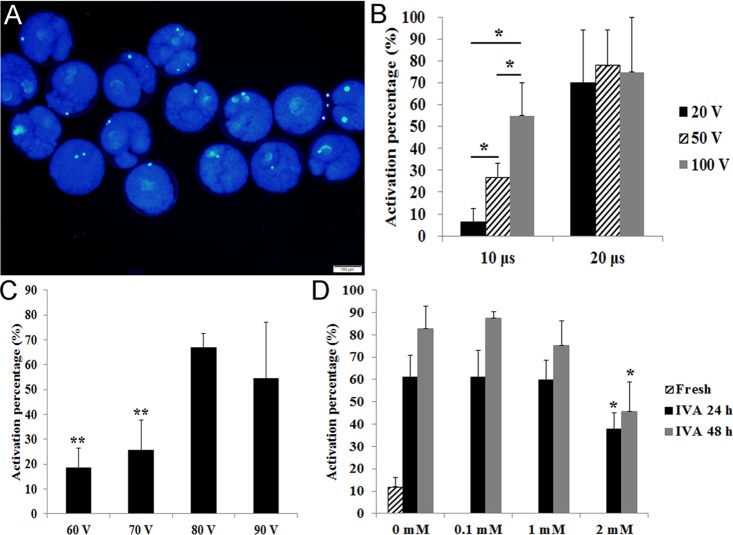
Effects of different stimulus conditions and concentrations of melatonin on oocyte activation (**A**) Fresh oocytes were activated by weak stimulus (70V, 10μs, 2 pulses and stained with DAPI to show female pronucleus formation. (**B**) Oocytes were activated by different combinations of electric pulse times (10 μs and 20 μs) with voltage intensity (20 V, 50 V and 100 V). (**C**) Oocytes were activated by different voltage intensity treatment (60 V, 70 V, 80 V and 90 V; 10 μs). (**D**) Oocyte aged for 24 hr or 48 hr *in vitro* were treated with different concentrations of melatonin and activated by weak stimulus set 70 V, 10 μs, 2 pulses. All graphs show mean ± s.e.m. Abbreviations used in this and all subsequent figures: IVA, *in vitro* aging. Independent replicates were conducted with a minimum of 25 oocytes/replicate, at least 3 stable replicates were obtained. *P<0.05, **P <0.01. Bar = 100 μm.

In our studies, oocytes with a female pronucleus or fragmented oocytes were defined as activated (Fig. 1A). The optimal condition for stimulation is very important to detect the degree of aging in oocytes. Few fresh oocytes and most aged oocytes should be activated under this condition. When we compared different stimulation times (10 μs VS 20 μs), we found that most fresh oocytes were activated under different voltages with 20 μs. However, fresh oocytes showed different degrees of activation under different voltages with 10 μs (Fig. 1B). To detect the optimal voltage for activation, we used 60 V, 70 V, 80 V and 90 V with 10μs to stimulate fresh oocytes and found that fresh oocytes had lower activation under 60 V or 70 V (Fig. 1C). So we used 70V and 10 μs to activate oocytes in the following experiments.

It has been reported that 1 mM melatonin could significantly inhibit oocyte aging in the mouse [9]. Here we used 0.1 mM, 1 mM and 2 mM melatonin to treat porcine oocytes for 24 hr or 48 hr. The results showed that 0.1 mM and 1 mM melatonin had no effects on oocytes aged for 24 hr or 48 hr *in vitro*. However, 2 mM melatonin could significantly decrease the activation percentage of aged oocytes after treatment for either 24 hr or 48 hr (Fig. 1D).

### Melatonin maintained normal oocyte morphology during postovulatory oocyte aging

**Figure 2:**
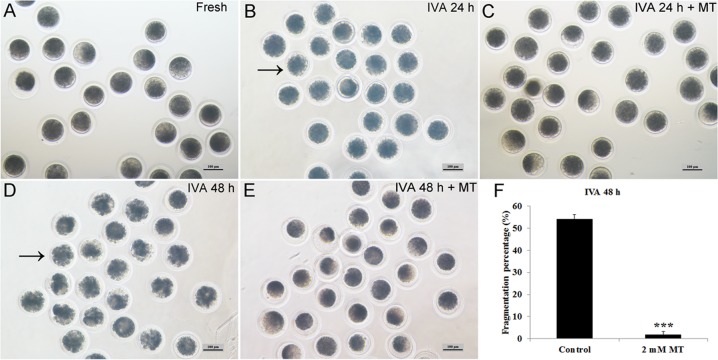
Effects of oocyte aging and melatonin on oocyte morphology (**A**) Fresh oocytes. (**B**) Oocytes aged for 24 hr. (**C**) IVA 24 hr oocytes treated with 2mM melatonin. (**D**) Oocytes aged for 48 hr. (**E**) IVA 48 hr oocytes treated with 2mM melatonin. (**F**) Fragmentation percentage in oocytes aged for 48 hr and treated with 2mM melatonin for 48 hr. Oocytes with abnormal morphology were indicated by arrowheads. All graphs show mean ± s.e.m. Abbreviations used in this and all subsequent figures: MT, melatonin. Independent replicates were conducted with a minimum of 30 oocytes/replicate, at least 3 stable replicates were obtained. ***P <0.001. Bar = 100 μm.

Melatonin could maintain oocyte morphology during postovulatory aging and decrease their spontaneous parthenogenetic activation (Fig. 2A-E). In oocytes aged for 24 hr *in vitro*, most of the oocytes showed normal morphology with low fragmentation and melatonin had no effects on them. However, the cytoplasm was non-uniform and it was dispersed being dark-pigmented in oocytes aged for 48 hr and more than half of the aged oocytes became fragmented (Fig. 2D, F). If oocytes were treated with 2 mM melatonin for 48 hr, this abnormal morphology could be inhibited effectively and the fragmentation percentage was decreased significantly to a very low percentage (Fig. 2E, F). This phenomenon was rarely presented in IVA 24 hr oocytes, as most of them still exhibited normal morphology (Fig. 2B) with an extremely low fragmentation percentage and there was no difference when they were treated with 2mM melatonin (Fig. 2C).

### Melatonin promoted embryonic development of postovulatory aging oocytes after parthenogenetic activation

**Figure 3:**
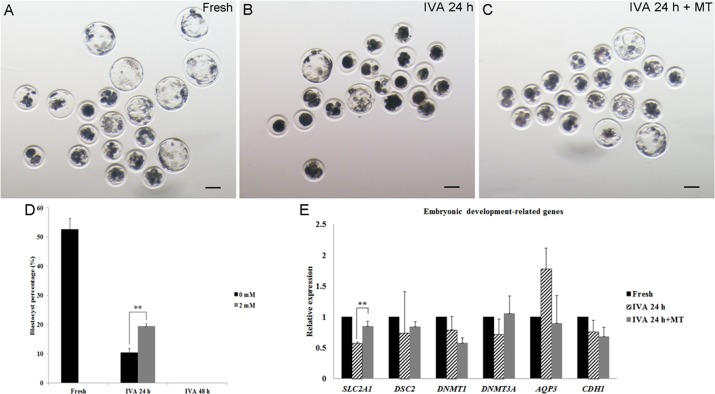
The development of embryos from fresh, aged and melatonin treated oocytes after parthenogenetic activation (**A**) Fresh oocytes. (**B**) Oocytes aged for 24 hr *in vitro*. (**C)** IVA 24 hr oocytes treated with 2mM melatonin. (**D**) Blastocyst formation from fresh oocytes, IVA 24 hr or 48 hr oocytes and oocytes treated with melatonin for 24 hr or 48 hr after strong stimulus (800V, 40μs, 2 pulses). (**E**) The expression of embryonic development-related genes (*SLC2A1*, *DSC2*, *DNMT1*, *DNMT3A*, *AQP3* and *CDH1*) in parthenogenetic blastocysts from fresh and aged oocytes. All graphs show mean ± s.e.m. Independent replicates were conducted with a minimum of 25 embryos/replicate, at least 3 stable replicates were obtained. **P <0.01. Bar = 100 μm.

In order to determine whether melatonin could maintain the quality of aged oocytes, especially regarding the subsequent embryonic development, parthenogenetic activation was carried out. Approximately 50% fresh oocytes could develop to the blastocyst stage (Fig. 3A, D), while only 10% blastocysts obtained from oocytes aged for 24 hr and none of the oocytes aged for 48 hr could develop to blastocysts after activation (Fig. 3B, D). In the presence of 2 mM melatonin, there were nearly 20% oocytes aged for 24 hr that could develop to blastocysts after parthenogenetic activation (Fig. 3C, D). However, melatonin had no impact on embryonic development of IVA 48hr oocytes and none of the activated oocytes could develop to blastocysts (Fig. 3D). To detect the effects of melatonin on the development of activated oocytes aged for 24 hr, we selected several embryonic development-related genes (*SLC2A1, DSC2, DNMT1, DNMT3A, AQP3* and *CDH1*) and measured their expression. We found that oocyte aging reduced the expression of *SLC2A1* in blastocysts from IVA 24 hr oocytes and melatonin could increase its expression significantly (Fig. 3E).

### Melatonin alleviated oxidative stress in postovulatory aging oocytes

**Figure 4:**
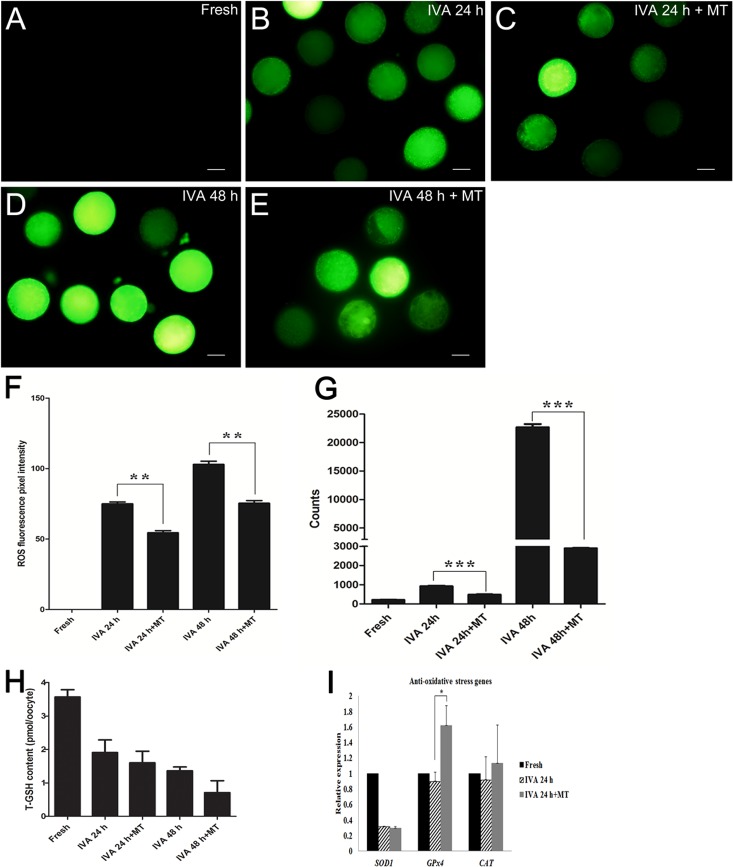
Effects of melatonin on ROS levels and total glutathione in aged oocytes (**A**) Fresh oocytes. (**B**) Oocytes aged for 24 hr. (**C**) IVA 24 hr oocytes treated with 2 mM melatonin. (**D**) Oocytes aged for 48 hr. (**E**) IVA 48 hr oocytes treated with 2 mM melatonin. (**F**) ROS fluorescence pixel intensity in IVA oocytes. (**G**) ROS level (counts of photons) detection of single cell by optical nanoprobes. (**H**) Total glutathione (T-GSH) content in fresh and IVA oocytes. (**I**) The expression of anti-oxidative stress genes (*SOD1*, *GPx4* and *CAT*) in fresh and aged oocytes. All graphs show mean ± s.e.m. Abbreviations used in this and all subsequent figures: ROS, reactive oxygen species. Independent replicates were conducted with a minimum of 30 oocytes/replicate, at least 3 stable replicates were obtained. **P <0.01, ***P <0.001. Bar = 50 μm.

Oxidative stress is one of the major challenges threatening the developmental potential, thus we used both ROS Assay Kit and single living cell detection system to perform the ROS detection assay. As shown in Fig. 4A-G, the ROS levels of aged oocytes are significantly higher than those of melatonin-treated oocytes either aged for 24 hr or 48 hr, which demonstrated that melatonin reduced oxidative stress during oocyte aging.

Because glutathione (GSH) has been shown to be present in various amounts in a diverse variety of cells and because it exerts powerful functions as an antioxidant in defending cells from the damage of oxidative stress, quantification of intracellular GSH was conducted. Surprisingly, the results showed that the level of intracellular GSH decreased significantly in aged oocytes and melatonin had no effects on increasing the level (Fig. 4H). We detected the expression levels of anti-oxidative stress genes (*SOD1, GPx4* and *CAT*) in oocytes aged for 24 hr. Remarkably, oocyte aging decreased the expression of anti-oxidative stress genes; however, melatonin could increase the expression of *GPx4* in IVA 24 hr oocytes (Fig. 4I).

### Melatonin decreased early apoptosis levels in postovulatory aging oocytes

**Figure 5:**
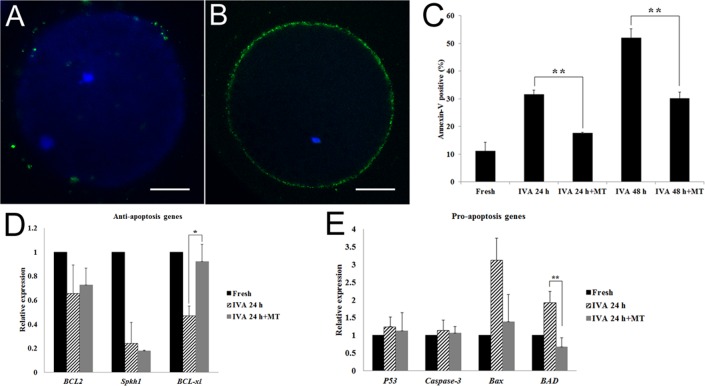
Effects of melatonin on early apoptosis in aged and melatonin treated oocytes by Annexin-V FITC detection assay (**A**) Negative control. (**B**) Apoptosis positive. (**C**) Apoptosis positive percentage in fresh, aged and melatonin treated oocytes. (**D)** The expression of anti-apoptosis genes (*BCL2*, *Sphk1* and *BCL-xl*) in fresh and aged oocytes. (**E**) The expression of pro-apoptosis genes (*P53*, *Caspase-3*, *Bax* and *BAD*) in fresh and aged oocytes. All graphs show mean ± s.e.m. Independent replicates were conducted with a minimum of 25 oocytes/replicate, at least 3 stable replicates were obtained. **P <0.01. Bar = 25μm.

It had been shown that oocyte aging is accompanied by cellular apoptosis; thus, we next detected early apoptosis levels in aged oocytes by the Annexin V-FITC assay. With this detection, the observation of a green circle indicating the location on the external cellular membrane of the oocyte was defined as Annexin V positive (Fig. 5B), otherwise it would be defined as negative (Fig. 5A). The results showed that only 11% oocytes displayed early apoptosis in fresh oocytes. This percentage increased to 32% and 52% if oocytes were aged for 24 hr or 48 hr *in vitro*. However, melatonin could inhibit early apoptosis during oocyte aging. The early apoptosis percentage in aged oocytes decreased to 18% and 30% significantly after treatment with melatonin for 24 hr or 48 hr (Fig. 5C).

Furthermore, we detected the expression of anti-apoptosis genes (*BCL2, Sphk1* and *BCL-xl*) and pro-apoptosis genes (*P53, Caspase-3, Bax* and *BAD*) in oocytes aged or treated with melatonin for 24 hr *in vitro*. For the anti-apoptosis genes, it was shown that their expressions were decreased in oocytes aged for 24 hr and melatonin could inhibit the decrease of the expression of *BCL-xl* (Fig. 5D). For the pro-apoptosis genes, oocyte aging induced the increase of *Bax* and *BAD*’s expression and melatonin could decrease their expression, especially *BAD*’s expression (Fig. 5E). This result suggested that melatonin blocked early apoptosis effectively during oocyte aging by regulating the expression of anti-apoptosis genes or pro-apoptosis genes.

### Melatonin retarded the decline of mitochondrial membrane potential ΔΨm in postovulatory aging oocytes

**Figure 6:**
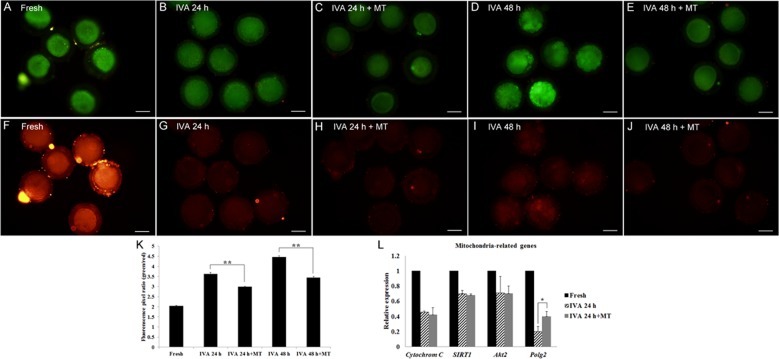
Effects of melatonin on mitochondrial membrane potential in aged and melatonin treated oocytes by JC-1 detection assay (**A**) Green-Fresh oocytes. (**B**) Green-Oocytes aged for 24 hr. (**C**) Green-IVA 24 hr oocytes treated with 2mM melatonin. (**D)** Green-Oocytes aged for 48 hr. (**E**) Green-IVA 48 hr oocytes treated with 2mM melatonin. (**F**) Red-Fresh oocytes. (**G**). Red-Oocytes aged for 24 hr. (**H**) Red-IVA 24 hr oocytes treated with 2 mM melatonin. (**I**) Red-Oocytes aged for 48 hr. (**J)** Red-IVA 48 hr oocytes treated with 2 mM melatonin. (**K)** Fluorescence pixel ratio (green/red) in fresh and aged oocytes. (**L**) The expression of mitochondria-related genes (*Cytochrome C*, *SIRT1*, *Akt2* and *Polg2*) in fresh and aged oocytes. All graphs show mean ± s.e.m. Independent replicates were conducted with a minimum of 25 oocytes/replicate, at least 3 stable replicates were obtained. **P <0.01. Bar = 50 μm.

It is well known that mitochondria play a crucial role in maintaining critical metabolic cell functions. So we carried out the mitochondrial membrane potential assay to investigate the mitochondrial membrane potential state during oocyte aging. To detect the mitochondrial membrane potential, we analyzed the ratio of green/red fluorescence. Clearly, fresh oocytes showed the lowest value (Fig. 6A, F, K); the aged oocytes displayed much higher ratios compared to those treated with melatonin for 24hr or 48hr, respectively (Fig. 6B-E, G-J, K), suggesting a significant efficacy of melatonin on keeping mitochondrial membrane potential in postovulatory aging oocytes. In addition, RT-qPCR was performed to quantify the expression levels of mitochondria-related genes (*Cytochrom C, SIRT1, Akt2* and *Polg2*) in oocytes aged for 24 hr. The results showed that oocyte aging decreased the expression of mitochondria-related genes; however, melatonin could increase some genes’ expression, like *Polg2* (Fig. 6L).

## DISCUSSION

The process of postovulatory oocyte aging is regulated by a series of molecular mechanisms that decrease the quality of oocytes and subsequent embryo development after fertilization. It has been reported that numerous functional gene products involved in cellular metabolism, reactive oxygen species and cell cycle regulation are differentially expressed during oocyte aging by using the Gene Ontology Analysis [31]. In this study, we used porcine oocytes as a model to demonstrate that melatonin could act as a powerful inhibitor to delay oocyte aging by maintaining normal morphology, reducing oxidative stress levels both on molecular and cellular levels, and therefore promoted embryonic development following successful activation.

We knew that a weak stimulus can artificially produce a relatively high activation percentage of aged oocytes compared to a relatively low activation percentage of fresh oocytes [32]. We found that 70V was an optimal voltage intensity in our studies and 2mM melatonin supplementation in culture media showed a dramatically reduced activation rate in IVA 24hr and IVA 48hr oocytes compared with control groups, which is similar to the result obtained by Lord et al [9]. The blastocyst percentage of melatonin-treated IVA 24 hr oocytes was nearly twice compared to that of untreated ones. However, nearly none of the IVA 48 hr oocytes or melatonin-treated IVA 48 hr oocytes developed to the blastocyst stage. The reason was that IVA 48hr oocytes were already “fully aged” and could not be rescued, and therefore the obvious observation of extremely high fragmentation rates in IVA 48 hr oocytes (Fig 2D, F). These results were consistent with the data reported by Kikuchi et al [32] and Miao et al [33]. We have reported that centrosomes in porcine oocytes aged for 48 hr were absent and spindles became abnormal and disorganized. Here, we found that melatonin could sharply decrease the fragmentation rates to an extremely low level in IVA 48 hr oocytes and displayed similar morphological phenotypes as seen in fresh oocytes (Fig. 2F), which strongly demonstrated that melatonin was an powerful free radicals scavenging compound to eliminate oxidative stress. We also found that melatonin could significantly increase the expression of *SLC2A1* (*solute carrier family 2 member 1*) in IVA 24 hr oocytes. *SLC2A1* is known as *GLUT1* (*Glucose transport 1*) and it is down-regulated in porcine oocytes because of blocked glucose transport in aged porcine oocytes [34]. A report revealed that low concentrations of melatonin supplied to fertilized embryos significantly up-regulated the expression of the developmentally important gene *SLC2A1* in bovine [35], and all these results prove that melatonin can promote the embryonic development of aged oocytes.

One of the most severe challenges that oocytes can encounter is oxidative stress, which contributes to oocyte aging. Given that Reactive Oxygen Species (ROS) production is directly associated with oxidative stress, we used both the ROS Assay Kit and a fluorescent probe capable of detecting single cells to perform ROS detection assays. We found that the ROS levels of aged oocytes was significantly higher than those of melatonin-treated aged oocytes either aged for 24 hr or 48 hr, which demonstrated that melatonin reduced oxidative stress in postovulatory aged oocytes. Furthermore, it is remarkable that the expression of *GPx4* (*glutathione peroxidase 4*) increased in the melatonin-treated group of IVA 24 hr oocytes (Fig 4I). As GPx4 was essential for mouse development and proved as a vital factor in protecting cells from oxidative damage [36], our data proved that melatonin may alleviate oxidative stress in postovulatory aged oocytes by enhancing the expression of the anti-oxidative stress gene *GPx4*. On the other hand, as an intrinsic antioxidant existing in oocytes, the content of glutathione (GSH) was expected to decrease with the progression of oxidative stress. However, our results showed that melatonin had no effect on increasing the content of GSH in aged oocytes (Fig 4H).

Oxidative stress is accompanied with cellular apoptosis; phosphatidylserine (PS) on the external cellular membrane is a marker for early apoptotic mature oocytes. Annexin V has a high affinity with PS, so we used Annexin V-FITC Apoptosis Detection kit to detect early apoptosis in aged oocytes. The result showed that melatonin could decrease early apoptosis levels in aged oocytes, which agreed with Lord’s report [9]. Both *BCL-xl* (*B-cell lymphoma-extra-large*) and *BAD* (*BCL2 Associated Agonist of Cell Death*) belong to the BCL-2 family. BCL-xl protein decreases apoptosis by controlling caspase activation, while the BAD protein functions opposite of *BCL-xl* [37]. In our studies, we found that melatonin increased the expression of BCL-xl and decreased the expression of *BAD* in aged oocytes, which suggested that melatonin blocked early apoptosis effectively during oocyte aging by regulating the expression of anti-apoptosis genes or pro-apoptosis genes.

Mitochondria play a crucial role in maintaining cellular metabolic functions, so we carried out the mitochondrial membrane potential assay to investigate the mitochondrial membrane potential state. A fluorescence probe, JC-1, tends to accumulate in the matrix of mitochondria, forms into J-aggregates and produces red excitation light when mitochondrial membrane potential is maintained high; if mitochondrial membrane potential is maintained low, JC-1 cannot accumulate in the matrix of mitochondria, hence forms into a monomer and generates green excitation light. In this assay, we found that melatonin had significant efficacy in keeping mitochondrial membrane potential in a normal state in postovulatory aged oocytes. Furthermore, melatonin increased the expression of *Polg2* (*mitochondrial-specific DNA polymerase gamma*) in IVA 24 hr oocytes and *Polg2* was proved to play important roles in mitochondrial replication and biogenesis [38] and normal mammalian embryogenesis [39]. It was reported that postovulatory oocyte aging was involved in ROS-induced mitochondrial injury [40] and the opening of the mitochondrial permeability transition pores (PTPs) [41], thus mtDNA replication would be interrupted and subsequent energy demand exceeded the possible support.

Taken together, our study demonstrated that melatonin effectively maintained the morphology, alleviated oxidative stress, decreased early apoptosis levels, retarded the decline of mitochondrial membrane potential in postovulatory aged oocytes and significantly promoted subsequent embryonic development. Compared to other antioxidants which had been reported to have disadvantages or limited potency [42], melatonin had more advantages because of its rapid metabolic rate and it caused less harm to oocytes. These results provide important information that could potentially be used to control oocyte aging in other animal species or human oocytes processed for clinical assisted reproductive technology.

## MATERIALS AND METHODS

Chemicals and reagents used in the present study were purchased from Sigma Chemical Co. unless otherwise specified.

### Preparation of porcine oocytes

Porcine ovaries were obtained from a slaughterhouse and transported to the laboratory while maintained at <34°C. Follicular fluid from 3–6 mm antral follicles was aspirated with an 18-gauge syringe. Cumulus oocyte complexes (COCs) with uniform cytoplasm and several layers of cumulus cells were selected and rinsed three times in washing medium (TCM-199 medium supplemented with 10% porcine follicular fluid (pFF), 5 μg/mL insulin, 10 ng/mL EGF, 0.6 mM cysteine, 0.2 mM pyruvate, 25 μg/mL kanamycin). Approximately 30 COCs per well were cultured in 96 well plates containing TCM-199 medium supplemented with 10% porcine follicular fluid (pFF), 5 μg/mL insulin, 10 ng/mL EGF, 0.6 mM cysteine, 0.2 mM pyruvate, 25 μg/mL kanamycin and 5 IU/mL of each eCG and hCG, covered with mineral oil. The oocytes were matured for 44 hr at 38.5°C, 5% CO_2_ in humidified air.

### *In vitro* aging of porcine oocytes

For oocyte aging *in vitro*, cumulus cells were removed by vortexing for 4 min in 0.1% hyaluronidase (in TLH-PVA [25], TL-Hepes medium supplemented with 0.1% PVA) after porcine COCs maturation for 44 hr. Only oocytes with first polar bodies were used for the experiments. The treated oocytes were then cultured in wells of a 96-well culture plate containing 150 μl of NCSU23 medium (108.7 mM NaCl, 4.8 mM KCl, 1.7 mM CaCl_2_.2H_2_O, 1.2 mM KH_2_PO_4_, 1.2 mM MgSO_4_.7H_2_O, 25.1 mM NaHCO_3_, 5.5 mM Glucose, 1.0 mM L-Glutamine, 7.0 mM Taurine, 5.0 mM Hypotaurine, 0.05 mg/mL Gentamicin, 4.0 mg/mL Fatty acid-free BSA) [26] and covered with mineral oil at 38.5°C under 5% CO_2_ in humidified air for 24 or 48 hr. For melatonin treatment, 100 mM melatonin stock was diluted to final concentration 2mM. The treated oocytes were cultured in wells containing 150 μl of freshly prepared culture medium described above and covered with mineral oil at 38.5°C under 5% CO2 in humidified air for 24 or 48 hr.

### Parthenogenetic activation and *in vitro* culture of embryos

Fresh or aged oocytes were placed between 0.2-mm-diameter platinum electrodes 1 mm apart in activation medium. Activation was induced with two direct-current (DC) pulses of 1.2 kV/cm for 40 μs on a BTX Elector-Cell Manipulator 200 (BTX, San Diego, CA) according to the experimental design. The medium used for activation was 0.3M mannitol, supplemented with 1.0 mM CaCl_2_, 0.1 mM MgCl_2_, and 0.5 mM Hepes. The orientation of oocytes and polar bodies was not vertical to platinum wire electrodes during electrical activation. After activation treatment, embryos were washed and transferred into NCSU medium with 5 μg/ml cytochalasin B for 4 hr to inhibit second polar body extrusion, then cultured in 150 μl NCSU medium covered with mineral oil in a 96-well culture plate. The culture environment was 5% CO_2_ in air at 38.5°C. Parthenogenetically activated oocytes were evaluated for the percentage of blastocysts on Day 6.

### RNA isolation and real-time RT-PCR

Total RNA was isolated from 50 porcine oocytes or blastocysts using RNA prep Pure Micro Kit (TIANGEN, Beijing, China). Enhanced GFP (eGFP) cRNA was transcribed *in vitro* from pIVT-eGFP [27] and 1 ng was added to each sample prior to RNA isolation as an internal control. Real-time RT-PCR was performed as previously described [28], using cDNA from two oocytes or embryos per reaction. Relative gene expression was calculated using the ΔCt method [29] with eGFP expression for normalization. Primers are listed in Table S1.

### Analysis of ROS levels

To identify oxidative stress/intracellular reactive oxygen species (ROS) levels in aged oocytes, a Reactive Oxygen Species Assay Kit was utilized to detect ROS as green fluorescent signals of DCFH diacetate (DCHF-DA; Beyotime Institute of Biotechnology, China). In brief, 20-30 oocytes from each treatment group were incubated at 38.5°C for 30 min in D-PBS containing 10 μM DCHF-DA. Then, the oocytes were washed 3 times in D-PBS containing 0.1% BSA before mounting on glass slides using D-PBS for microscopy. The fluorescence intensity in each oocyte was measured under a fluorescence microscope (Olympus, Tokyo, Japan) with the same scan settings for each sample. The fluorescent images were saved as graphic files in TIFF format. The normal fluorescence pixel intensities of each oocyte were analyzed using ImageJ software (version 1.50; National Institutes of Health, Bethesda, MD, USA).

To confirm the above results, we also detected ROS levels in single fresh and aged oocytes by employing optical fiber probes with ~10 μm tips (Rayme, Wuxi, China). The distal end of the tip was designed free for transmitting light by careful adjustment of the fiber placement angle in the sputter chamber. The silver coating of the nanoprobes is stable for subsequent fabrication procedures. The counts of photons were constantly detected and calculated during a 150-s period following the tips inserted into an immobilized oocyte in a PBS droplet [30].

### Detection of intracellular GSH

The concentrations of total glutathione (T-GSH) were examined with a GSH/GSSG assay kit (Beyotime Institute of Biotechnology, China) based on an enzymatic method according to the manufacturer’s instructions. A total of 30-40 oocytes from each group were mixed with 30 μl of protein scavenger M solution supplied by the kit and vortexed thoroughly for 5 min, then the mixture was frozen at -80°C for 2 min and thawed at 38.5°C repeatedly for three times. The mixture was centrifuged at 10,000 rpm for 10 min at 4°C and put on ice for 5 min. The absorbance was monitored continuously at 412 nm with a microplate spectrophotometer (PerkinElmer, USA) for 25 min, with a reading recorded every 5 min and regarded the readings at the time point 25 min as the final absorbance values.

### Annexin V-FITC assay

According to manufacturer’s instructions, Annexin V–fluorescein isothiocyanate (FITC) staining was performed using an Annexin V-FITC Apoptosis Detection kit (Vazyme, Nanjing, China) to detect the externalization of phosphatidylserine (PS) in early apoptotic MII oocytes. Briefly, 20-30 MII oocytes were washed twice in PBS containing 0.01% PVA (w/v), then incubated in 100μl binding buffer containing 5μl of Annexin V-FITC for 10 min at room temperature in the dark, transferred to 100μl 4% paraformaldehyde (PFA) for fixation for 1hr after being washed twice in PBS containing 0.01% PVA (w/v); after fixation and washes twice in PBS containing 0.01% PVA (w/v) again, oocytes were mounted on glass slides using DAPI for DNA fluorescence microscopy. Annexin-V positive oocytes were identified under a confocal system (Zeiss LSM 510 META, Germany) with 450–490 nm (excitation) and 520 nm (emission) filters.

### Determination of mitochondrial membrane potential

The mitochondrial membrane potential of the oocytes was evaluated using a mitochondrial membrane potential assay kit with JC-1 (Beyotime Institute of Biotechnology, China). Oocytes were exposed to 10μM JC-1 in 100μl working solution at 38.5°C in 5% CO_2_ for 20 min, after which they were washed with washing buffer to remove surface fluorescence, and then mounted on glass slides using D-PBS for microscopy. Laser excitation was set at 488 nm for green and 525 nm for red fluorescence, respectively. The fluorescence intensity in each oocyte was measured under a fluorescence microscope (Olympus, Tokyo, Japan) with the same scan settings for each sample. The fluorescent images were saved as graphic files in TIFF format. The normal fluorescence pixel intensities of each oocyte were analyzed using ImageJ software (version 1.50; National Institutes of Health, Bethesda, MD, USA). The ratio of green to red fluorescence pixels was used to analyze mitochondrial membrane potential.

### Data analysis

For each treatment, three replicates were performed. Statistical analyses were carried out by analysis of variance. Differences between control, IVA groups and melatonin treated groups were evaluated by independent-sample t-tests using SPSS 22.0 statistical software. Data are expressed shown as mean ± SEM and P< 0.05 is considered as significant.

## SUPPLEMENTARY MATERIAL


