# Supplementary material for: Melatonin prevents postovulatory oocyte aging and promotes subsequent embryonic development in the pig
Source: Aging (Albany NY). 2017 Jun 26;9(6):1552–63. doi: 10.18632/aging.101252 (PMC5509455; doi:10.18632/aging.101252)
Supplement: Supplementary file 1 [file aging-09-1552-s001.pdf]

SUPPLEMENTARY MATERIAL

Table S1 Primer sequences for RT-qPCR.

Gene	Primer sequence 5'-3'	Access No./reference	Length (bp)
BCL2	F: TTCTTTGAGTTCGGTGGGG	XM_003121700.4	195
	R: CCAGGAGAAATCAAATAGAGGC		
Sphk1	F: CTACGAGCAGGTGACGAATG	XM_005656916.2	132
	R: AGGCTGAGCACGGAGAAGAC		
BCL-x1	F: ACTGAATCAGAAGCGGAAAC	Liu et al. [1]	294
	R: AAAGCTCTGATACGCTGTCC		
P53	F: CTTTGAGGTGCGTGTTTGTG	NM_213824.3	122
	R: TGGGCAGTGCTCGCTTA		
Caspase-3	F: GCCATGGTGAAGAAGGAAAA	Li et al. [2]	132
	R: GGCAGGCCTGAATTATGAAA		
Bax	F: GGTCGCGCTTTTCTACTTTG	Li et al. [2]	111
	R: CGATCTCGAAGGAAGTCCAG		
BAD	F: TTTGAGCAGAGTGAGCAGGAA	XM_003122573.4	218
	R: TCTGGGTAAGAGCTGTGGC		
CytoChrom C	F: GAAAAGGGAGGCAAACACAA	Li et al. [2]	117
	R: CCAGGTGATGCCTTTGTTCT		
SIRT1	F: TTGATCTTCTCATTGTTATTGGGTC	Li et al. [2]	62
	R: ACTTGGAATTAGTGCTACTGGTCTTA		
Akt2	F: GAGGTGCTGGAGGACAACGA	XM_013988560.1	82
	R: CGCCGCACATCATCTCGTA		
Polg2	F: GGCTTGATTTCTGGCTACG	Li et al. [2]	166
	R: ACATTCCACAGGGTTTCTATT		
SOD1	F: TCCATGTCCATCAGTTTGGA	Li et al. [2]	131
	R: AGTCACATTGGCCCAGGTCTC		
GPx4	F: ATTCTCAGCCAAGGACATCG	Li et al. [2]	93
	R: CCTCATTGAGAGGCCACATT		
CAT	F: ACATGGTCTGGGATTCTGG	Li et al. [2]	99
	R: TCATGTGCCTGTGCCATCT		
SLC2A1	F: GGTGCTCCTGGTCCTGTTCT	XM_013977359.1	125
	R: CGGGTGTCTTGTCGCTTT		
DSC2	F: GTGAAAGGAGGGCACCAGA	XM_005674194.1	174
	R: ACGGGGCTGCGTGTAACTGT		
DNMT1	F: CTACCTGGCTAAAGTCAAATCCC	XM_005654829.2	117
	R: TCACCACTTGTCTGGCTCCC		
DNMT3A	F: GGACAAGAATGCCACCAAATC	XM_005662686.2	185
	R: CGAACCACATGACCCAACG		
AQP3	F: TGGCTATGCCGTCAACCCT	NM_001110172.1	218
	R: GCTTCACATTCTCCTCGTCAGT		
CDH1	F: TAATGATGTGGCACCAACCC	NM_001163060.1	156
	R: TCGTAATCAAACACCAGCAGAG		

SUPPLEMENTARY REFERENCES

1. LIU J, WANG Q-C, HAN J et al. Aflatoxin B1 is toxic to porcine oocyte maturation. Mutagenesis 2015; 30:527-535.

2. LI Y, ZHANG Z, HE C et al. Melatonin protects porcine oocyte in vitro maturation from heat stress. Journal of pineal research 2015; 59:365-75.
